# Development and evaluation of a dual density insole for people standing for long periods of time at work

**DOI:** 10.1186/s13047-020-00402-2

**Published:** 2020-07-08

**Authors:** Jennifer Anderson, Anita E. Williams, Chris Nester

**Affiliations:** School of Health and Society, University of Salford, Salford, M5 4WT UK

**Keywords:** Footwear comfort, Standing, Shoes, Insoles, Arch height, Customisation, Personalisation, Occupation, Workplace

## Abstract

**Background:**

Appropriate footwear is important for those who stand for prolonged periods of time at work, enabling them to remain comfortable, healthy and safe. Preferences for different footwear cushioning or hardness are often person specific and one shoe or insole will not be the choice for all. The aim of this study was to develop a range of insole options to maintain comfort during long periods of standing at work and test insole material preferences in the workplace.

**Methods:**

The study consisted of two parts. Part one evaluated 9 insoles of the same geometry that varied in hardness under 2 different plantar regions (*n* = 34). Insole preference, plantar pressure and selected anthropometric foot measures were taken. Three insole designs based on the most preferred options were identified from this part.

In part two, these three insoles were evaluated with 22 workers immediately after trying them on (1 min) and after a working day. Foot anthropometric measures and subjective questions concerning material hardness preferences and self-reported foot characteristics were used to investigate whether either had a relationship with insole preference.

**Results:**

Part one found insole preference predominantly varied according to material hardness under the medial arch rather than the heel/forefoot. Softer material under the heel and forefoot was associated with a reduction in peak pressures in these regions (*p* < 0.05). The most preferred insole had lower pressures under the hallux and first metatarsal phalangeal joint, and greater pressures and contact area under the medial midfoot (p < 0.05) compared to the least preferred insole. Height and foot anthropometrics were related to insole preference.

In part two, under real world conditions, insole preference changed for 65% of participants between the immediate assessment (1 min) and after a whole workday, with dorsum height related to the latter (*p* < 0.05). Subjective questions for self-assessed arch height and footwear feel identified 66.7% of the insole preferences after 1 day at work, compared to 36% using immediate assessment of insole preference.

**Conclusion:**

Preference for material hardness varies underneath the medial arch of the foot and is time dependent. Simple foot measures and questions about comfort can guide selection of preferred insoles.

## Background

Footwear comfort is extremely important for workers spending prolonged periods of time on their feet [1–3]. Uncomfortable shoes may be rejected by workers and replaced by their own alternatives [2], potentially compromising safety in some work settings. The importance of footwear comfort is further emphasised by the relationship between footwear comfort, preference and musculoskeletal injury risk [4–6].

Although footwear comfort is subjective, complex and affected by multiple physical and psychological factors, preferred footwear can be quickly identified when a shoe is tried on [7–9]. Previous research considering footwear comfort has largely focused on running [8–10] or military recruits [5, 11]. The specificity of these populations and the associated physical activity limits the transferability to understanding footwear for workers who stand for long periods of time. Furthermore, studies of shoe and insole design frequently vary in more than one variable (e.g. changes in both insole geometry and materials), preventing an understanding of the effect of each independent design variable on footwear comfort.

Despite the limitations of previous research, material hardness, or cushioning, has been identified as a dominant factor influencing footwear comfort in running and military populations [5, 12, 13]. Qualitative research with working populations, including those undertaking prolonged standing, identify self-reported links between footwear comfort and footwear cushioning [1, 2], suggesting that material hardness is also important for these populations. Although softer insoles have been associated with greater comfort scores [10, 11], insoles rated as less comfortable by the majority are still the preferred choice for other individuals [4, 5, 14]. Indeed, our previous research identified preference variations between individuals for footwear sole hardness over 3 hrs of standing [15]. This strongly suggests that comfort and cushioning preferences are person specific. Therefore, to improve comfort, footwear solutions may need to include variations in cushioning that the wearer can choose for themselves.

For workplace footwear and online footwear purchases, it is often not possible to try footwear on and allow wearers to explore their comfort preferences. As such, other methods that might allow a person to select their preferred choice without trying any shoes on are desirable. Objective measures have been associated with footwear comfort, including person specific biomechanical variables such as plantar pressure, joint kinematics, joint kinetics, and muscle activity [8, 14], as well as body and foot arch height, foot and leg alignment, and foot sensitivity [5, 15].

Although it is not possible for a lay person to evaluate their own foot characteristics to a level comparable to a health or research professional, simple factors such as concepts of foot arch height have the potential to provide an indication of a measure since they can be visually assessed. Self-assessment could lead to improved selection of preferred footwear at the point of sale, especially for online sales. Therefore, we also consider the relationship between footwear preference and pragmatic self-assessments of foot measurements that could be presented in an online tool with no training requirements.

This study aimed to investigate the impact of insole material preferences on footwear comfort, focussing on the specific needs of those involved in long periods of standing at work. The work comprised two parts:

1. To investigate the impact of variations in heel/forefoot and medial arch material hardness on insole comfort, plantar pressure and its relation to wearer characteristics to inform the development of a range of insoles.
2. To test the developed insoles from part one in a real-world setting and investigate the ability of wearer characteristics and subjective questions to predict the selection of the preferred insole.

## Part 1: methods

Aim: To investigate the impact of variations in heel/forefoot and medial arch material hardness on insole comfort, plantar pressure and its relation to wearer characteristics to inform the development of a range of insoles.

### Participants

Thirty-four healthy participants (male: 14, female: 20) aged 18–55 years and with shoe size UK 5–9 were recruited from a University population. Ethical approval and individual written consent were gained prior to testing.

### Footwear

**Fig. 1:**
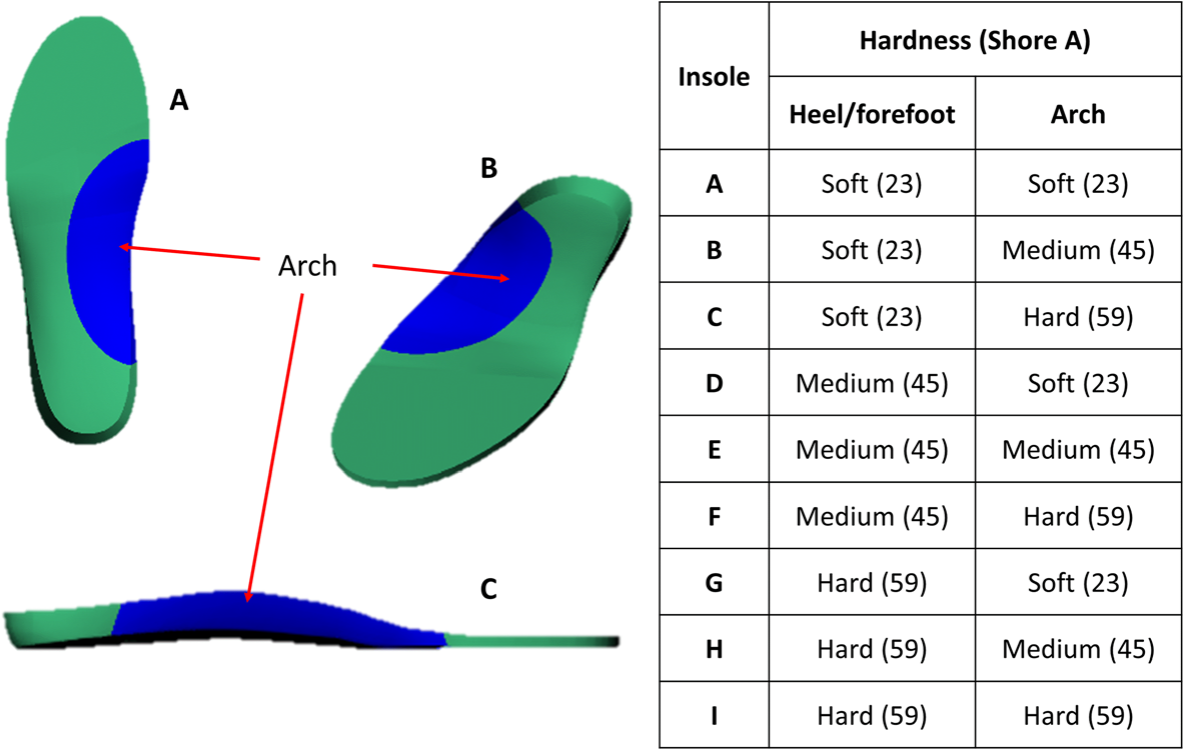
Insole from bottom (**a**), top (**b**) and medial view (**c**) and hardness combinations for each insole

**Fig. 2:**
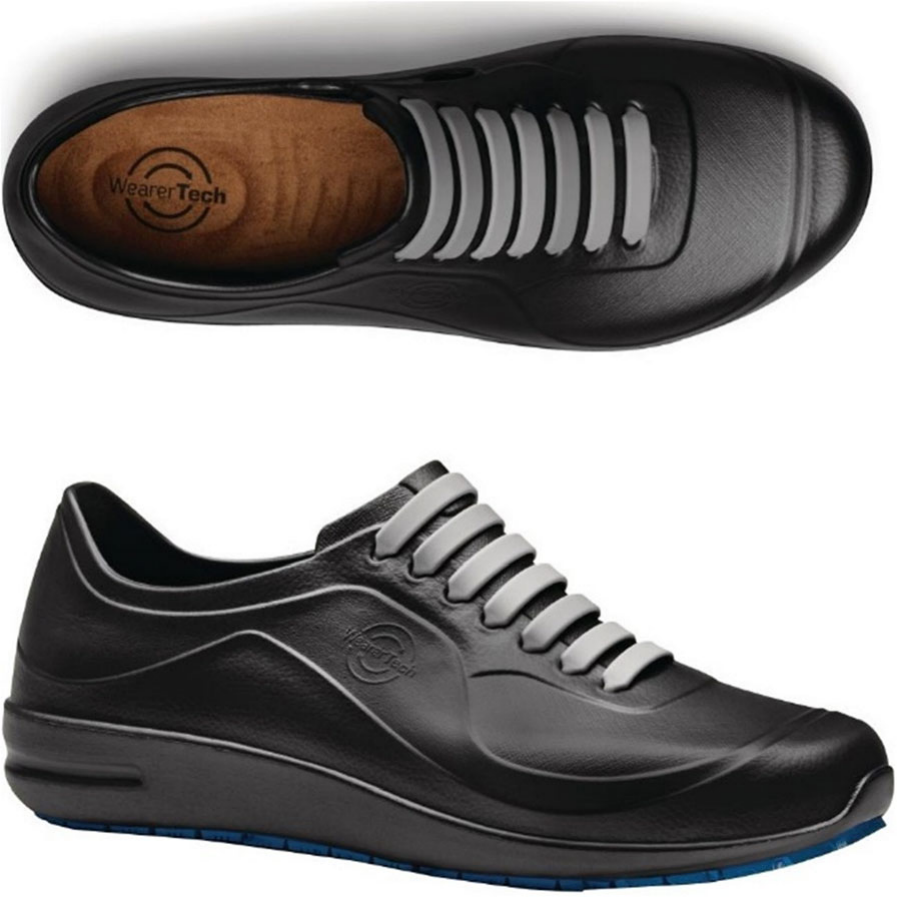
Shoe used for part 1. The entire shoe is made from EVA with a slip resistant sole. The interior of the shoe has no arch shape so all underfoot contouring is from the inserted insole

Nine different insoles were produced, varying only in hardness. The insole was a minimum of 5 mm thick and had a contoured medial arch based on the profile of a current product with an in-built arch shape (EziKlog, WearerTech). Each insole comprised two parts, a heel/forefoot piece and a medial arch piece (that was secured to the heel/forefoot piece, Fig. 1). Three different EVA materials were used for each section: soft, medium and firm (Shore A: 23, 45 and 59), creating 9 different insoles. A microfiber layer covered the top and bottom of the insole to maintain its integrity. The insole was designed to fit a work shoe made from EVA (Energise, WearerTech, Fig. 2).

### Protocol

Participants were recruited from a University population, none of who worked in occupations requiring prolonged standing and were excluded if they had any current lower limb injuries. Participant characteristics were measured during bilateral standing (Table 1).

**Table 1:** Measures taken in part 1 and 2 of study

Measurement Variable	Equipment	Part 1	Part 2
Age		•	•
Height	Stadiometer	•	•
Weight	Scales	•	•
Foot Length	Brannock Device	•	•
Foot Width	Brannock Device	•	•
Foot Arch Length	Brannock Device	•	•
Q angle	Goniometer	•	
Dorsal height at 50% foot length	Digital Calliper [16]	•	•
Height of Navicular Tuberosity	Digital Calliper	•	
Height MTPJ1	Digital Calliper	•	
Height MTPJ5	Digital Calliper	•	
Heel Width	Callipers	•	
Ball of foot circumference	Gulick II tape measure	•	•
Short heel circumference	Gulick II tape measure	•	
Foot Posture Index	[17]	•	•
Foot mobility magnitude	Digital Calliper [16]		•
Plantar Pressure	Pedar-X	•	

There were 10 conditions presented in a randomised order, one for each of the developed insoles and one randomly selected insole that was tested twice to assess the repeatability of the comfort measure. Participants were blind to the insole differences and unaware that there was a repeat condition.

Participants were asked to wear a comfortable pair of their own shoes for a control walk (≈20 m) that took place prior to each of the 10 test conditions. This process created a “washout” effect, because footwear worn prior to a test can impact comfort ratings [18] and we sought to standardise this for the different test conditions. Participants were handed the test shoe with the insole already inside. Thin socks were worn throughout. Between trials participants sat down for the duration of each footwear change either side of the control walk. They were told they could pause for a longer break if required at any point, although this was not required by any participant.

Three work-based movement tasks were undertaken in each condition: a walk up and down the room (≈20 m), one static standing task (screwing nuts onto bolts) and a dynamic standing task (hitting coloured targets on the corners of a 150x40cm desk according to instructions set out on a laptop screen in front of the participant). Both standing tasks were completed at a desk 0.9 m high, lasted 1 min each and aimed to simulate work-like standing tasks. Instructions were given to keep feet on the floor and not to rest weight on the desk surface.

At the start of the protocol, participants were advised to note down any thoughts regarding the comfort of the condition after each individual insole had been worn to help them to rank the insoles once testing was complete. This note taking was at their own discretion. At the end of the session, when all insoles had been worn, the insoles were ranked from 1 (most preferred) to 10 (least preferred).

Plantar pressure data was collected for 23 of the 34 participants, using the Pedar-X (Novel GmbH, Germany) system operating at 50 Hz. A target sample size of 20 had been identified based on similar research and prioritising plantar pressure data [14, 16]. However, our advertising led to over recruitment (*n* = 34) and we decided to only use plantar pressure data we needed (albeit with 3 extra data sets) so as to reduce participant burden while also taking advantage of the added value that the more subjective measures of comfort necessarily require (i.e. *n* = 14 extra data sets).

### Data analysis

Plantar pressure data was analysed using Matlab (2016b). Pressure data was cropped to remove steps associated with gait initiation, cessation and turning. Single strides were identified using a 5 kPa threshold for heel strike and toe off. Standing tasks were broken into four 15 s epochs with average values calculated for each task. For all data, the foot was divided into 9 regions: the whole foot, heel, medial midfoot, lateral midfoot, metatarsal phalangeal joints 1 (MTPJ1), 2–3 (MTPJ2–3), 4–5 (MTPJ4–5), hallux and lesser toes. The following variables were calculated for each area: mean pressure, peak pressure and percentage contact area. Contact area was defined as the area covered by sensors registering a pressure of greater than 5 kPa [15]. Average values of the left and right feet together were taken for the steps of each participant and for the 4 epochs in each standing task.

All statistical analysis was completed using SPSS (v23, IBM). To assess the differences in plantar pressure between task and insole, a two-way repeated measures ANOVA with a Bonferroni post hoc correction was used. Task (walk vs static standing vs dynamic standing) and insole (A-F) were the two independent variables. Differences in plantar pressure variables between the preferred and least preferred insole were tested with one-way between subject ANOVAs with Bonferroni post hoc correction with task (walk vs static standing vs dynamic standing) and insole (preferred vs least preferred) as the independent variables. One-way between subject ANOVAs and independent t-tests identified differences in participant characteristics (height, weight, foot measurements) when they were grouped according to their preferred insole. Comparisons were made between any insole that was the preferred choice of 5 or more participants (A vs B vs C vs F); those that had a preference for a soft or medium heel/forefoot section (A, B, C vs D, E, F); and those that had a preference for a soft, medium or hard arch (A, D, G vs B, E, H vs C, F, I). Prior to statistical analysis, the FPI data was converted into its Rasch transformed score to enable parametric analysis [19]. A Friedman test was used to determine any differences in the ranking of the insoles. For post hoc tests, a Wilcoxon rank tests with Bonferroni corrections resulting in an adjusted significance level of *p* < 0.0014.

## Part 1: results

Participant characteristics are presented in Table 2. Insoles with the soft heel/forefoot section (A, B and C) were the most preferred, being ranked number 1 by 32, 21 and 21% of participants respectively, and therefore 74% of participants in total (Table 3). In the arch area, 42% preferred a soft, 24% a medium and 36% a firm material. There was a statistically significant effect of the ranking of the 9 insoles (χ^2^(8) = 36.893, *p* < 0.001) with post hoc tests finding insole A ranked significantly lower than insoles G and I (i.e. was more preferred), and insole B ranked lower than insole G.

**Table 2:** Participant characteristics for part 1 (*n* = 34). Absolute refers to direct measurements whereas normalised refers to measurements normalised to foot length

Variable		Mean	SD	Min	Max
Age (years)		31.6	10.4	18	54
Height (m)		1.69	0.06	1.54	1.81
Weight (kg)		70.7	12.6	55	99
BMI (kg/m^2^)		24.8	4.8	19.2	37.9
UK Shoe Size		7	2	5	9
Q angle (°)		8.6	2	5.5	12.5
FPI		3.9	2.7	−1	8
Foot Length (mm)		252.1	9.5	236.5	276.5
Arch Length	Absolute (mm)	182.2	7.1	173	199.5
	Normalised (%)	72.3	1.4	70.0	77.3
Foot Width	Absolute (mm)	92.4	4.8	84.5	102.5
	Normalised (%)	36.7	1.8	33.5	40.4
Dorsal Arch Height	Absolute (mm)	61.8	5.7	51.5	72.0
	Normalised (%)	24.5	2.1	20.6	28.3
Navicular Height	Absolute (mm)	43.8	6.6	28.5	57.0
	Normalised (%)	17.3	2.5	11.5	22.1
MTPJ1 height	Absolute (mm)	34.0	3.7	20.0	39.5
	Normalised (%)	13.5	1.4	8.1	15.5
MTPJ5 height	Absolute (mm)	23.0	2.3	19.0	30.0
	Normalised (%)	9.1	0.9	7.7	11.5
Heel Width	Absolute (mm)	58.2	5.2	46.5	70.0
	Normalised (%)	23.1	2.1	18.6	27.1
Ball of foot Circumference	Absolute (mm)	236.7	15.2	209.5	289.5
	Normalised (%)	93.9	5.7	85.5	114.7
Heel circumference	Absolute (mm)	276.5	11.9	255.5	300
	Normalised (%)	109.7	3.8	103.0	117.7

*SD* Standard deviation;* FPI* Foot Posture Index

**Table 3:** Ranking of insoles for part 1 where a ranked position of 1 indicates the most preferred insole and 9 the least preferred. N.B. totals add to 101% due to errors caused by rounding to whole numbers

		% of individuals (*n* = 34)								
	Insole	A	B	C	D	E	F	G	H	I
	Heel/forefoot	Soft	Soft	Soft	Medium	Medium	Medium	Firm	Firm	Firm
	Arch	Soft	Medium	Firm	Soft	Medium	Firm	Soft	Medium	Firm
Ranked Position	1	32	21	21	6	3	15	3	0	0
	2	9	26	15	18	15	3	6	6	3
	3	21	12	9	6	9	18	9	9	9
	4	3	15	6	18	24	6	0	18	12
	5	9	3	15	12	3	12	15	12	21
	6	9	6	9	12	21	15	6	9	15
	7	3	3	0	18	12	21	18	21	6
	8	6	6	12	6	15	6	21	6	24
	9	9	9	15	6	0	6	24	21	12

The insole that was repeated was used to assess the reliability of the ranking process. The average difference in ranking position for the same insole was 3.125, where a difference of 1 would mean they had been ranked next to each other. In total, 38% of individuals had a difference of 1 rank, 37% had a difference of between 2 and 4 while the remaining 25% had a difference of between 5 and 7. This likely reflects comments made by a few participants about the difficulty of ranking some of the insoles. When comparing the plantar pressure between preferred insoles, a comparison was only made between the highest and lowest ranked insole (1st and 9th) as this was always larger than the difference in ranking for the repeated insole case.

### Plantar pressure

The average number of steps analysed for each insole was 30 ± 3 and there was no meaningful difference of insole on foot contact time (F_5,91_ = 0.669, *p* = 0.635), a representative measure of walking speed [20]. All data is shown in Table 3.

Peak pressure for the whole foot, heel, medial midfoot, MTPJ1, hallux and toes, increased as the heel/forefoot piece got harder (Table 4). In the lateral midfoot, insole A (soft heel/forefoot, soft arch piece) had significantly lower pressure than insoles C, F and H (all with hard/medium arch pieces). The MTPJ1 region displayed significantly lower pressures for insole A (soft heel/forefoot, soft arch piece) than D (medium heel/forefoot, soft arch piece) and H (firm heel/forefoot, medium arch piece). In the MTPJ2–3 and toe regions there were lower pressures in insole C (soft heel/forefoot, firm arch piece) than insole D and E (medium heel/forefoot, soft and medium arch pieces). Contact area differences were seen for the lateral and medial midfoot only. In the medial midfoot, the greatest contact area was seen for insoles B and C (soft heel/forefoot, medium/firm arch piece) with lowest values seen for insole D (medium heel/forefoot and soft arch piece) and for insoles with the firm heel/forefoot insole sections (G, H, I).

**Table 4:** Plantar pressure differences between insoles (p < 0.05). MP = mean pressure (kPa); PP = peak pressure (kPa); CA = contact area (%). Arrows indicate significant post hoc differences between insoles. (↓ = insole has values less than …; ↑ = insole has values greater than …). ^s^ = significantly different to static standing value, ^y^ = significantly different to dynamic standing value, ^w^ = significantly different to walking value. ^a^ significant interaction effect for insole and task

Region	Variable	Insole									Mean			
		A	B	C	D	E	F	G	H	I	Walking	Static Standing	Dynamic Standing	
Whole foot	MP										32.23^sy^	22.10^wd^	21.22^ws^	
	PP	↓EFGHI	↓DEFGHI	↓EFGHI	↓GHI↑B	↓GHI↑AB	↓GHI↑ABC	↑ABCDEF	↑ABCDEF	↑ABCDEF	296.82^sy^	96.83^wy^	143.04^ws^	^a^
	CA										51.88^s^	59.19^wy^	53.92^s^	
Heel	MP										42.96^sy^	32.27^w^	33.91^w^	
	PP		↓DEGHI	↓GHI	↑B			↑B	↑BC	↑B	216.13^sy^	83.50^wy^	113.0^ws^	^a^
	CA										54.91^sy^	69.39^w^	68.25^w^	
Lateral midfoot	MP	↓CFH		↑A			↑A		↑A		28.86	27.79^y^	26.00^s^	
	PP										112.99^sy^	60.71^wy^	90.57^ws^	
	CA	↓H							↑A		57.89^sy^	75.29^wy^	66.68^ws^	
Medial midfoot	MP	↑DGHI	↑DFGHI	↑DFGHI	↓ABCEFH	↑DG	↓BC	↓ABCE	↓ABC↑D	↓ABC	15.19	16.19	14.96	
	PP	↑GH	↑DGHI	↑DGI	↓BC	↑G	↑G	↓ABCEF	↓AB	↓B	83.69^sy^	45.53^wy^	63.45^ws^	
	CA	↓C↑DG	↑DGI	↑A	↓ABCE	↑DG	↓C	↓ABCE	↓C	↓BC	37.42^sy^	51.05^wy^	44.19^ws^	^a^
MTPJ 1	MP	↓DH		↓D	↑AC					↑A	46.15^sy^	26.63^wy^	21.18^ws^	
	PP	↓GHI	↓DGHI	↓GI	↑B		↓GHI	↑ABCF	↑AB	↑ABCF	210.42^sy^	66.03^wy^	100.27^ws^	^a^
	CA										63.86	67.99^y^	58.16^s^	^a^
MTPJ 2–3	MP			↓DE	↑C	↑C					41.98^sy^	22.58^wy^	19.06^ws^	
	PP			↓DEFGHI	↑C	↑C	↑C	↑C	↑C	↑C	200.54^sy^	53.58^wy^	92.83^ws^	
	CA										64.60^y^	64.74^y^	55.18^ws^	
MTPJ 4–5	MP										27.91^sy^	19.33^wy^	17.92^ws^	
	PP										148.48^sy^	50.64^wy^	85.59^ws^	
	CA										54.26	58.13	51.70^s^	
Hallux	MP										38.09^sy^	17.87^w^	16.94^w^	^a^
	PP	↓GH	↓EGHI	↓EFGHI	↓GHI	↑BC	↑C	↑ABCD	↑ABCD	↑BCD	258.19^sy^	48.89^wy^	106.88^ws^	^a^
	CA										52.68	52.59^y^	46.79^s^	
Toes	MP			↓DE	↑C	↑C					41.14^sy^	23.10^wy^	19.57^ws^	
	PP		↓EH	↓DEGHI	↑C	↑BC		↑C	↑BC	↑C	200.21^sy^	23.85^wy^	94.46^ws^	^a^
	CA										63.27	65.96^y^	56.54^s^	

Comparing the most and least comfortable insoles (Table 5), the preferred insole had greater medial midfoot mean pressure (+ 22%), peak pressure (+ 16%) and contact area (+ 15%) compared to the least preferred. Whole foot peak pressure was on average 22% lower for the preferred insole compared to the least preferred. Peak pressure was 19 and 18% lower for the MTPJ1 and hallux in the preferred insole compared to the least preferred. A significant interaction between task and insole hardness occurred as a result of a much greater differences between insoles during walking.

**Table 5:** Significant plantar pressure differences between the preferred and least preferred insole. Percentage change is the difference from the least preferred insole to the preferred (i.e. a negative % difference indicates the value is reduced in the most preferred)

Region	Variable	Most preferred (Mean kPa)	Least Preferred (Mean kPa)	% difference	F	P	Interaction effect
Whole Foot	PP	158.83	204.75	−22%	26.94	< 0.001	^a^
Hallux	PP	125.02	152.18	−18%	7.83	0.01	^a^
MTPJ1	PP	115.94	142.71	−19%	12.52	0.002	^a^
Medial Midfoot	CA	50.19	43.58	+ 15%	10.07	0.004	^a^
	MP	18.07	14.81	+ 22%	10.37	0.004	^a^
	PP	72.39	62.6	+ 16%	4.89	0.038	

^a^ significant interaction effect for insole and task. *PP* peak pressure, *CA* contact area, *MP* mean pressure

### Individual wearer characteristics

There was a significant main effect of arch length on insole preference (F_3,29_ = 3.05, *p* = 0.047) with a greater absolute arch length (*p* = 0.041) in those who preferred insole F (medium heel/forefoot, firm arch) in comparison to insole A (soft heel/forefoot, soft arch).

Compared to those preferring a soft heel/forefoot section, those that chose a medium heel/forefoot section were taller (t_18.6_ = 2.9, *p* = 0.009, soft heel/forefoot = 1.67 ± 0.06 m; medium heel/forefoot = 1.73 ± 0.04 m) and had a greater absolute arch length (soft = 180.1 ± 5.5 mm; medium = 188.3 ± 8.0 mm, t_10.8_ = 2.85, *p* = 0.016).

A smaller normalised heel width was present in those who preferred the harder arch piece (F_2,33_ = 3.43, *p* = 0.045) with post hoc results finding a greater normalised heel width in those preferring the soft arch compared to the hard arch (soft arch = 23.9 ± 1.6%; hard arch = 21.7 ± 1.9%, *p* = 0.044). Although not significant, there was a trend towards a greater FPI score (lower arched feet) in those preferring the firm arch piece (F_2,33_ = 2.57, *p* = 0.093).

## Part 2: methods

Aim: To test the developed insoles from part one in a real-world setting, and investigate the ability of wearer characteristics and subjective questions to predict the selection of the preferred insole.

### Participants

Participants were all kitchen workers (*n* = 22), selected because our previous research demonstrates that they are spending an average 87% of work time on their feet, of which around ¾ is spent performing standing tasks [21]. Exclusion criteria included anyone under the age of 18, anyone who did not work back of house in the kitchen or was not on their feet for most of the day and anyone with diagnosed foot conditions or lower limb injuries.

### Footwear

**Fig. 3:**
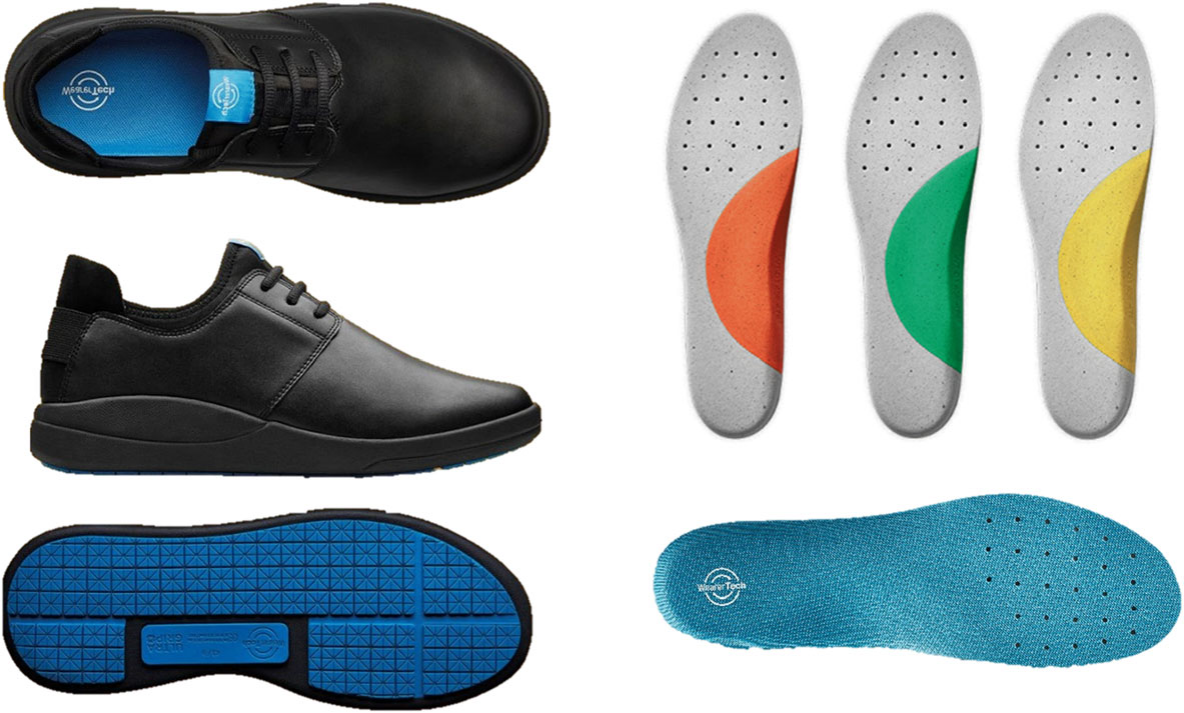
Insoles and shoe given to kitchen workers (part 2)

Based on the outcomes of part one, three insoles were developed, all made from EVA with the same contouring and fabric top cover (Fig. 3). All had a soft heel/forefoot section of Shore A 30, but the arch piece was either soft (Shore A 30), medium (Shore A 40) or firm (Shore A 50). This choice reflected the results of part 1 where preference predominantly varied about the insole arch hardness, with most participants preferring a soft heel/forefoot section. This was supported by the reduction in peak plantar pressure values under the heel and forefoot associated with the softest material.

Each participant was given all 3 insoles and a lace-up shoe suitable for their workplace setting (‘Relieve Custom Pro’, WearerTech, Fig. 3), with an EVA midsole, slip resistant rubber outsole, a microfibre upper and neoprene stretch lining.

### Protocol

Participant characteristics were measured during bilateral standing (Table 1). These measures were chosen pragmatically based on time available, which was limited with the working population, and factors that individuals would potentially be able to provide an indication of themselves. Each participant tried the shoe on with each of the 3 insoles in a randomised order (approximately 1 min per insole), blind to the differences between insoles. They were told they could walk around and assess the comfort dynamically but were not given specific instructions. They were asked to rank the insoles in order of preference. This process aimed to replicate how a shoe may be selected in a shop prior to purchase and provide an indication of immediate preferences.

After the initial testing session, participants completed 7 questions in an online survey that subjectively rated their own foot characteristics (Table 6). The link to the online questionnaire was sent once the researcher had left to ensure it was completed alone. This provided a means of exploring whether participants could independently evaluate their own feet in ways that could predict their preferred insole, mimicking the potential point of sale or circumstances when they might choose footwear without trying them on. This included questions regarding foot characteristics that have previously been related to footwear comfort, such as medial arch height, foot arch flexibility and foot sensitivity [5] as well as questions regarding their preference for material under the whole foot, arch of the foot and how supportive they liked a shoe to feel.

**Table 6:** Multiple choice questions for the self-assessment of foot characteristics by participants

Question		Answer Options
1	Please identify what type of foot arch you have when standing (image selector)	1. Low arch: When viewed from the sider there will be very little, if any arch shape to the foot, with no room to put a finger under the arch. Almost the entire sole of the foot will make contact with the ground causing the wet footprint to be filled in with very little narrowing in the band connecting the heel and forefoot.
		2. Medium arch: When viewed from the side there will be a visible arch from the heel to the ball of the foot with just enough room to fit an index finger under. In the wet footprint, the forefoot and heel will be visible but there will be an obvious narrowing in the band connecting them.
		3. High arch: When viewed from the side there will be a very visible arch. An index finger will be able to fit under the arch with room to move. In the wet footprint, the forefoot and heel will be very narrow or non-existent.
2	Do your feet look more arched when the weight is taken off them?	1. No, my feet look the same sitting and standing
		2. Yes, my feet get slightly more arch shape to them when I take the weight off them
		3. Yes, my feet get a lot more arch shape to them when I take the weight off them
3	How do you prefer a shoe or insole to feel under your foot?	1. Soft with less support
		2. Medium firmness with some support
		3. Firm material for more support
4	How do you prefer a shoe or insole to feel under the arch of your foot?	1. Soft with less support
		2. Medium firmness with some support
		3. Firm material for more support
5	How do you feel when walking barefoot on a pebbly beach?	1. Ouch! I really struggle to walk barefoot on a pebbly beach.
		2. Uncomfortable but I can manage.
		3. Not a problem, I’m happy to walk barefoot on a pebbly beach
6	How do you feel when walking barefoot on a hard floor (concrete or tiles)?	1. Ouch! I really struggle to walk barefoot on a hard floor.
		2. Uncomfortable but I can manage.
		3. Not a problem, I’m happy to walk barefoot on a hard floor
7	How sensitive are the soles/underside of your feet?	1. Not at all sensitive
		2. Slightly sensitive
		3. Very sensitive

The participants wore each insole (soft, medium and firm arch materials) in the shoe for an entire day at work in a randomised order. They were asked to ensure a similar length of time in each insole and to wear them on consecutive days at work. The time the insole had been worn was recorded at the end of each day. Once each insole had been worn for a full day at work, participants were asked to rank the insoles in order of preference.

### Data analysis

A Friedman test was used to assess the differences in rankings of the insoles. Prior to statistical analysis, the FPI data was converted into its Rasch transformed score to enable parametric analysis [19]. One-way between subject ANOVAs were used to identify differences in characteristics between individuals that preferred each insole (soft arch vs medium arch vs firm arch). For the subjective questions answered by the participant, a chi squared test determined any relationship with the preferred insole (both immediate (1 min) preference and after one workday preference). As well as the FPI total score, the analysis was also completed with the score for ‘height and congruence of the medial lateral arch’ due to the similarity between this measure and the visual assessment of arch height. Subjective questions with a relationship to insole preference of *p* < 0.25 were used to identify the preferred insole.

## Part 2: results

The length of workday (i.e. time wearing each insole) varied from 7 to 16 h between individuals (average: 9.4 ± 2.8 h) but individual participants wore each insole for a similar length of time (i.e. the person with a working day of 7 h wore each insole for 7 h). This is reflected in the fact that there was no overall difference in the length of time each insole was worn for the entire group (*p* > 0.05). Participant characteristics can be seen in Table 7. Two participants did not complete the protocol so were removed from the analysis, one due to a job change and contact was lost with the second.

**Table 7:** Participant characteristics for part 2 (*n* = 20). Absolute refers to direct measurements whereas normalised refers to measurements normalised to foot length

		Mean	SD	Min	Max
Age (years)		30	7.9	20	53
Height (m)		1.72	0.09	1.56	1.90
Weight (kg)		78.8	18.5	51.7	126.6
BMI (kg/m^2^)		26.7	5.2	19.7	39.5
UK Shoe Size		8	2	4	12
FPI		2.7	2.8	0	11
Foot Mobility Magnitude		1.4	0.4	0.7	2.1
Foot Length (mm)		257.7	15.8	231.0	289.5
Arch Length	Absolute (mm)	188.5	13.1	168.5	217.0
	Normalised (%)	73.1	1.2	71.3	75.2
Foot Width	Absolute (mm)	96.3	6.0	83.0	106.5
	Normalised (%)	37.4	1.6	34.1	40.0
Dorsal Arch Height	Absolute (mm)	63.7	5.1	55.7	72.3
	Normalised (%)	24.6	1.8	21.6	28.0
Ball of foot Circumference	Absolute (mm)	241.2	16.0	214.5	267.0
	Normalised (%)	93.6	3.28	85.8	98.7

*SD* standard deviation; *FPI* foot posture index

### Insole preference

The Friedman test identified no overall difference between the rankings of the insoles (p > 0.05). In total, 65% of participants changed their insole preference between the immediate assessment after 1 min of wear and after wearing each insole for one day at work.

### Individual characteristics

There was no relationship between initial insole choice and any of the measured characteristics in Table 1 (p > 0.05). For the preferred insole choice after one workday, the only variable related to insole preference was dorsum height as a percentage of foot length (F_2,16_ = 4.221, *p* = 0.034). Those preferring the insole with a softer arch had a greater dorsum height (soft = 26.3 ± 1.1; medium = 24.4 ± 1.4; hard = 24.0 ± 1.7).

All but one participant rated their arch height as low or medium, therefore high arch was removed from the statistical analysis and an independent t-test was used in place of the ANOVA. Independent t-tests found a significant difference in dorsum height (as a percentage of foot length) between those rating their own arch as “low” compared to “medium” (t_16_ = 2.136,*p* = 0.048; CI = 0.014–3.77) and a trend towards a greater FPI score (i.e. a lower arch) in those with a self-reported low arch (Table 8).

**Table 8:** Difference in arch height between self-assessed arch heights of low and medium (only one person selected a high arch, so they were removed from analysis)

	Low Arch		Medium Arch		P value
	Mean	SD	Mean	SD	
Absolute Dorsum height (mm)	61.3	7.2	64.5	5.0	0.309
Dorsum height (% foot length)	23.4	2.1	25.3	1.4	0.048
FPI score	3.6	2.1	1.6	1.8	0.063

### Subjective questions

For the immediate insole preference, questions 5, 6 and 7 regarding foot sensitivity and question 4 (‘How do you prefer a shoe or insole to feel under your foot?’) had *p* values below 0.25. As all the sensitivity questions assessed the same factor, only the question ‘How do you feel when walking barefoot on a hard floor (concrete or tiles)?’ was included as it had the strongest association with insole preference. Using these two questions, the model could identify 68.4% of individuals immediate preference.

For the insole preference following a full workday, only questions 1 and 3 had a p value below 0.25. These were the self-assessment of arch height and the question ‘How do you prefer a shoe or insole to feel under your foot?’. These two questions could identify 66.7% of insole preferences after a whole working day. Based on these two questions a tool to assist a wearer to self-identify their preferred insoles was created (Table 9).

**Table 9:** Selection tool to determine insole preference after one workday. High arch column is based on only 1 inidividual reporting a high arch

		What type of foot arch do you have when standing?		
		Low arch	Medium arch	High arch
How do you prefer a shoe to feel under the arch of your foot?	Soft material with less support	Medium Insole	Soft Insole	Soft Insole
	Medium firmness with some support	Firm insole	Medium Insole	Soft Insole
	Firm material for more support	Firm Insole	Firm Insole	Medium Insole

## Discussion

This paper documents the research-led development of an insole product range that aims to improve the comfort of a shoe specific to workplace settings that demand prolonged standing. It identified that variation in preferences for material hardness was related mainly to the medial arch area (part one). Also, that most participants changed their footwear preference between the immediate assessment (1 min) and the assessment after wearing each insole for a whole workday (part two), highlighting the challenge of using first try on, or online purchases, in ensuring that preferred shoes are chosen. Finally, self-reported arch height and underfoot material preference were found to assist in the identification of the preference after a whole workday (part two), and thus there may be potential to use simple questions to guide footwear selection.

The preferred insole in part one had greater medial midfoot pressure compared to the least preferred insole and a resultant reduction of pressure in other regions. This is in agreement with previous walking and standing research [14, 15, 22, 23] and suggests that for these work-based tasks lower plantar pressure was an important component for comfort. Medial midfoot pressure was increased by using a softer material in the heel/forefoot section or by having a harder material under the medial arch. A softer material under the heel/forefoot presumably compresses more readily than the harder materials, thus allowing load to be transferred to the medial arch area. Indeed, the least preferred designs were those in which there was a softer material under the arch than in the heel/ forefoot section. This outcome also reveals that material choice alone can manipulate plantar pressure distribution and perceived comfort, independent of the much-discussed changes in insole geometry [24–26].

Only one participant selected an insole with a firm heel/forefoot section as their preferred choice in part one indicating that there is possibly a maximum hardness value above which comfort is less likely to be achieved. Furthermore, one study that tested insoles with a hardness of 52–75 shore A (similar to our hardest material) did not find a preference for insoles at the lower (softer) end of this range [27]. This perhaps suggests that above this maximum hardness value variations in hardness do not link to variations in comfort or preference. Defining the range of hardness values over which comfort and preferences vary could be important in personalising footwear options since it will offer genuine options for users to adjust their comfort and preferences.

Previous research has highlighted a preference for the entire insole to be harder for individuals with a lower medial arch height [5]. Based on our results this relationship is only true for the medial arch area, meaning a softer material can be used in other regions to improve comfort. The preference for harder arch materials in those with lower arched feet might be due to an increase in foot arch height that the material provides [28]. A softer arch material may enable contact with the arch in medium and high arched feet [29] and this contact may affect comfort [14, 15]. However, we did not measure the actual response of the arch geometry and foot joints to the insoles.

Due to the inability of most workers and online purchasers to try on footwear, methods that might allow a person to select their preferred insole without trying any shoes on or have objective measures taken are desirable. Results from this study suggest self-assessed arch height and preferred materials seem likely to enable a prospective wearer the opportunity to identify an insole that would be their preferred choice for longer-term use (e.g. after day at work). Based on the results, these two pieces of information would allow identification of the preferred insole in 67% of individuals, an improvement on using the immediate selection (36%). The difference in objective foot arch height measures between those rating themselves as having a low or normal arch suggests this measure could be a good indicator of arch height, but a larger study is warranted to verify this, especially focussing on the inclusion of more high arched participants.

The change in insole preference in part two following wear over a day at work (lasting 7–16 h) compared to a few minutes use could be a result of adapting to the insoles, changes in the feet due to prolonged standing, changes in the insoles over time, or a combination of these. For example, we know that prolonged standing causes changes in the pain pressure threshold [30], lower limb swelling [15, 31] and plantar pressure [15, 32]. In running, a reduction in arch height is also seen over time [32, 33]. Although this has not been assessed during prolonged standing, increases in medial midfoot pressures and contact area suggests that this might be the case [15]. Any changes in arch height or foot morphology from swelling could alter comfort and insole preference. This highlights the importance of developing a method to enable a user to identify the optimum footwear choice in the longer-term even if using only immediate assessments of their feet and footwear options.

In terms of the comfort assessment selection, ranking shoes has previously been shown to be the most reliable method of assessing comfort [9, 34], although the maximum number of footwear options that can be tested has not been reported. The use of ten different insoles in part one could have made it difficult to rank them, as noted by a few participants, and it assumes the difference between insoles are large enough to produce repeatable ranking. Although some studies suggest only using individuals who can rate comfort ‘reliably’ [34, 35], this may remove individuals with important characteristics, such as those with a low foot sensitivity, a factor that impacts insole preference [5]. This work took a more pragmatic approach because it had a specific target audience.

This study had several limitations. The variation in shoes used for part one and two was necessary as the shoe designed to accommodate the insoles was under development itself during part one, which is typical of real-world industry linked research. However, insoles were selected based on rankings and the footwear designs would have to dramatically affect rankings, not just comfort scores, to lead to different outcomes. While standardising the shoe for each part was important for the methodology, in a real-world context the use of a range of shoes means the transferability of these insoles to different shoes with varying midsole cushioning and geometries is unclear. Changing other aspects of the footwear may produce different results and interact with the insole effects we report here. The footwear development had commercial limitations, including the fact that the number of insole options was limited due to tooling and logistics costs. The number of participants recruited in part two of the study was limited by the number of pairs of sample shoes and insoles that the factory was willing to produce rather than developing recruitment numbers through a power analysis. Further research should test the self-assessment of arch height with a larger population, assess the accuracy of the subjective questions for identifying the preferred insole, and investigate changes in variables such as arch height and insole material properties over a working day. Considering the geometry (height and length) of the arch piece is also required.

Finally, we do not know the effect of wearing comfortable footwear on long term musculoskeletal disorders and injury risk at work. The comfort paradigm [4] suggests that a comfortable shoe might be one that is best for the body and therefore reduce lower body injury risk, as reported for military personnel and rugby players [5, 6]. We do not currently know if a similar protective effect would stem from comfortable footwear during prolonged standing at work, but if it impacted musculoskeletal disorders and overall discomfort, it could be beneficial for employers, employees and workplace safety policies.

## Conclusion

This study used preferred insole choices and plantar pressure data to enable the personalisation of insole choice for workers undertaking prolonged standing wearing specialist workplace footwear. There were differences in the preferences for insole material hardness between immediate assessment and assessment following one workday in each insole. The use of self-assessment of foot arch height and material preferences offers an opportunity to guide insole selection at the point of choosing footwear. The strength of this research lies in the practical application of data to the design and evaluation of new insole products that aim to protect prolonged standing workers.

## Data Availability

The datasets used and/or analysed during the current study are available from the corresponding author on reasonable request.

## Abbreviations

MTPJ: Metatarsophalangeal joint
SD: Standard Deviation
